# Euglycemic Diabetic Ketoacidosis With Acute Renal Failure: A Challenging Case for Clinicians

**DOI:** 10.7759/cureus.60171

**Published:** 2024-05-12

**Authors:** Rizwanullah, Tahera Ahmadi, Aftab Ahmad, Waqar Khan, Jose C Rosario-Curcio

**Affiliations:** 1 Internal Medicine, Hayatabad Medical Complex Peshawar, Peshawar, PAK; 2 General Practice, Sehat Hospital, Herat, AFG; 3 General Medicine, Cork University Hospital, Cork, IRL; 4 Medical School, University of Medicine and Health Sciences, San Juan, PRI

**Keywords:** oral antidiabetic drugs, fasting ramadan, fasting, challenging case, serum ketones, metabolic acidosis, urinary tract infection, acute renal failure, euglycemic diabetic ketoacidosis, diabetic ketoacidosis

## Abstract

Diabetic ketoacidosis (DKA) is a severe complication of diabetes mellitus characterized by hyperglycemia, metabolic acidosis, and ketosis. We present a challenging case of euglycemic DKA secondary to fasting and urinary tract infection with acute renal failure in a 50-year-old woman. Despite normal random blood sugar levels, the patient exhibited clinical signs of DKA, leading to further investigation. High anion gap metabolic acidosis with hyperkalemia and abnormal renal function tests were identified. After hemodialysis, serum ketones were found to be highly positive, confirming the diagnosis. Prompt management led to a complete clinical and laboratory resolution. This case underscores the importance of considering DKA in patients with suggestive symptoms, even with normal blood sugar levels.

## Introduction

Diabetic ketoacidosis (DKA) is a serious complication of diabetes mellitus (DM) and is the primary reason for hospital admissions, morbidity, and mortality in individuals with DM. DKA is characterized by hyperglycemic crises leading to metabolic acidosis, the production of ketoacids, dehydration, and electrolyte imbalances, potentially resulting in a diabetic coma [1]. However, DKA can present with euglycemia, which is defined as having blood glucose levels below 250 mg/dL. About 6% of patients exhibit blood glucose levels below 300 mg/dL, while approximately 1% of patients have levels below 180 mg/dL. The primary causes are insulin administration during transport to the hospital and fasting [2]. Dehydration, largely triggered by glucose-induced osmotic diuresis and vomiting, significantly contributes to acute kidney injury (AKI) in DKA patients. It is widely acknowledged that AKI has a considerable impact on long-term morbidity and mortality. AKI can manifest as mild increases in serum creatinine to anuric renal failure necessitating dialysis [3].

Here, we present a challenging case of euglycemic DKA (eu-DKA) secondary to fasting and urinary tract infection (UTI) with acute renal failure in a 50-year-old woman.

## Case presentation

A 50-year-old woman who had been managing hypertension and type 2 diabetes for 10 years with oral hypoglycemic medication (sitagliptin and metformin 50/1,000 mg twice a day) arrived at Hayatabad Medical Complex with symptoms including altered mental status, fever, bilateral flank pain, burning urination, and nausea and vomiting (multiple episodes) over the past two days. She had been fasting for the last 16 hours.

During the examination, her vital signs were as follows: temperature of 101°F, pulse rate of 104 beats/minute, respiratory rate of 24 breaths/minute, blood pressure of 110/80 mmHg, and oxygen saturation of 94% on room air. Further examination revealed pale conjunctiva, signs of dehydration, no yellowing of the eyes (scleral icterus), and no swollen lymph nodes. Her chest was clear bilaterally, with no abnormal sounds. In the abdomen, she exhibited tenderness in the bilateral flank regions, but there was no enlargement of the liver or spleen, rebound tenderness, or peripheral edema. On neurological examination, her Glasgow Coma Scale (GCS) score was 11/15, her pupils were of normal size and reacted to light, and there was no neck stiffness. Reflexes were normal. A cardiovascular examination did not reveal any abnormalities. Upon arrival, her random blood sugar was measured at 189 mg/dL. All investigations performed during her hospital stay are presented in Table 1.

**Table 1:** Lab investigations during hospital admission. WBC = white blood cell count; ALT = alanine transaminase; ALP= alkaline phosphatase; CRP = C-reactive protein; LDH = lactate dehydrogenase; pCO_2_ = partial pressure of carbon dioxide; HCO_3_ = bicarbonate ion

Labs	Reference range	Day 1	Day 2	Day 3	Day 4	Day 5
WBC (×10^3^/µL)	04–11	15	13	N/A	8	N/A
Hemoglobin (mg/dL)	11.5–7.5	6.5	9.2	10.5	11	N/A
Platelet count (×10^3^/µL)	150–450	415	338	385	315	N/A
Serum calcium (mg/dL)	08–10	8.4	N/A	N/A	N/A	N/A
Blood urea (mg/dL)	18–45	242	182	135	112	75
Creatinine (mg/dL)	0.42–1.06	4.5	3.04	2.54	1.98	1.3
Sodium (mmol/L)	135–150	164	144	138	137	135
Potassium (mmol/L)	3.5–5.1	6.5	5.5	3.4	3.6	3.8
Chloride (mmol/L)	96–112	122	116	99	106	93
Random blood sugar (mg/dL)	70–140	189	235	205	245	195
Total bilirubin (mg/dL)	0.1–1.0	0.1	N/A	N/A	N/A	N/A
ALT (IU/L)	10­50	15.4	N/A	N/A	N/A	N/A
ALP (IU/L)	35–104	64.3	N/A	N/A	N/A	N/A
CRP (mg/dL)	<0.5	3.6	N/A	N/A	N/A	0.9
Blood pH	7.35–7.45	7.25	7.35	7.28	7.38	7. 39
Blood HCO_3_ (mmol/L)	22–28	10	13	11	21	24
pCO_2_ (mmHg)	35–45	18	28	24	35	38
Anion gap (mmol/L)	04–12	32	15	28	10	8
Serum ketones (nmol/L)	<1.0	N/A	5.6	4.8	2.5	0.7
Urine pus cells	0–5	20–25	N/A	N/A	02–05	N/A
Urine output (mL/24 hours)	800–2,000	Nil	600	900	1,300	1,800
CPK (µg/L)	10–120	700	200	180	150	130
LDH (IU/L)	140–280	855	456	334	312	290
HbA1c (%)	<5.7	9	N/A	N/A	N/A	N/A

Upon discovering nearly normal glucose levels, we explored other potential diagnoses besides DKA. Renal function tests (RFTs) revealed elevated blood urea and creatinine levels, while serum electrolytes showed hypernatremia, hyperchloremia, and hyperkalemia. To stabilize the cardiac membrane, we administered 10 mL of 10% calcium gluconate, 10 units of insulin, and 100 mL of 25% dextrose intravenously. Both an electrocardiogram (ECG) and chest X-ray were normal. Arterial blood gases (ABGs) indicated metabolic acidosis with a high anion gap. As the patient had not produced urine in the past eight hours, we scheduled her for hemodialysis, which resulted in clinical improvement. Urine analysis revealed numerous pus cells, prompting us to add intravenous ceftriaxone 1 g twice daily to cover UTI, along with providing symptomatic management.

Due to low hemoglobin levels, we transfused two pints of red cell concentrate on days two and three. Repeat ABGs on the second day confirmed metabolic acidosis with a high anion gap, leading us to check serum ketone levels, which were high. Subsequently, we initiated the DKA protocol, adjusting fluid levels to prevent volume overload due to reduced urine output. By the fourth day of admission, the patient was clinically stable, and all investigations returned to normal by the fifth day.

Following a thorough investigation, we concluded that our case was eu-DKA with acute renal failure due to fasting and UTI, a rare and challenging condition for clinicians. Prompt diagnosis and management are crucial to avoid serious complications. We referred this case to the endocrinology department for proper dosing of oral hypoglycemic drugs and guidance on fasting while on antidiabetic medication.

## Discussion

DKA is a serious complication of both type 1 and type 2 diabetes. It manifests with hyperglycemia, ketosis, and an increased anion gap metabolic acidosis. These symptoms are typical of DKA due to either absolute or relative insulin deficiency. First reported in 1973, eu-DKA is characterized by ketoacidosis and electrolyte imbalances with minimal or no elevation of serum glucose, often below 11.0 mmol/L (200 mg/dL) [4]. Patients experiencing eu-DKA often display symptoms such as malaise, dyspnea, nausea, vomiting, and abdominal pain, which closely resemble those observed in conventional DKA. It is important to screen any diabetic patient with these symptoms for eu-DKA using tests for blood pH and blood or urine ketones. However, it is worth noting that eu-DKA may also be the initial presentation of diabetes. In eu-DKA, patients typically have normal blood glucose levels despite metabolic acidosis (pH below 7.3) and reduced blood bicarbonate levels (below 18 mmol/L). Elevated levels of serum and urine ketones are crucial for diagnosing eu-DKA [5].

Eu-DKA is mainly triggered by a carbohydrate deficit, resulting in heightened lipolytic activity and ketoacidosis. This deficit can be caused by factors such as glucose loss (from events such as glucosuria or vomiting) and reduced hepatic glycogen levels (from events such as starvation or chronic alcoholism). Patients with eu-DKA typically exhibit low serum insulin levels and an excess of counterregulatory hormones such as glucagon, cortisol, and adrenaline. A significant volume depletion occurs due to osmotic diuresis from glucosuria, often exacerbated by reduced oral intake and vomiting [5].

Acute renal failure is rare with DKA, especially in developing countries, as recently reported. Prerenal uremia, common in DKA, typically improves within a few hours with fluid therapy. Intrinsic renal failure, less common but with high mortality rates, is marked by increasing urea and creatinine levels and anuria. Underlying factors for acute renal failure in children with DKA include sepsis, shock, severe dehydration, rhabdomyolysis, and thrombotic microangiopathic syndrome. The incidence of acute renal failure varies from 3.7% to 11.5%. Management involves adjusting fluid and electrolyte therapy, insulin dosage, bicarbonate infusion, and considering renal replacement therapy such as hemodialysis or peritoneal dialysis. Mortality rates range from 40% to 72% in acute renal failure in DKA [6].

Initial management focuses on fluid resuscitation for severe dehydration. Intravenous crystalloid fluids should be administered until the resolution of the anion gap and acidosis. Unlike traditional DKA treatment, 5% dextrose should be included in fluid resuscitation to prevent hypoglycemia and expedite ketosis clearance. Switching to 10% dextrose can be considered if ketoacidosis persists despite 5% dextrose [5,7].

Our case presented with clinical signs and symptoms of DKA, but due to normal random blood sugar, we considered alternative diagnoses. This led to the identification of high anion gap metabolic acidosis with hyperkalemia and abnormal RFTs. After one session of hemodialysis, we tested for serum ketones, which returned highly positive. We then managed her as a case of eu-DKA secondary to fasting and UTI with acute renal failure. The patient was discharged with complete clinical and laboratory resolution. This case was particularly challenging because both DKA and uremia (renal failure) present with high anion gap metabolic acidosis, highlighting the importance for physicians to consider DKA even if a patient presents with normal random blood sugar but clinical symptoms indicative of DKA.

## Conclusions

Our case underscores the importance of considering DKA even in patients with normal random blood sugar levels but clinical symptoms suggestive of DKA. We presented a challenging case of eu-DKA secondary to fasting and UTI with acute renal failure in a 50-year-old woman. Despite normal random blood sugar levels, our patient exhibited typical signs and symptoms of DKA, prompting further investigation. Elevated anion gap metabolic acidosis, hyperkalemia, abnormal renal function tests, and positive serum ketones confirmed the diagnosis. We ruled out starvation and alcoholic ketoacidosis. Management involved fluid resuscitation, hemodialysis, antibiotic therapy for UTI, and adjustment of insulin therapy. The patient responded well to treatment, with complete clinical and laboratory resolution upon discharge. This case highlights the need for vigilance and thorough evaluation in diagnosing and managing eu-DKA, which can have serious complications if not promptly recognized and treated.

## References

[1] Guariguata L, Whiting DR, Hambleton I, Beagley J, Linnenkamp U, Shaw JE (2014). Global estimates of diabetes prevalence for 2013 and projections for 2035. Diabetes Res Clin Pract.

[2] Azkoul A, Sim S, Lawrence V (2022). Diabetic ketoacidosis in adults: part 1. Pathogenesis and diagnosis. South Sudan Med J.

[3] Chen J, Zeng H, Ouyang X (2020). The incidence, risk factors, and long-term outcomes of acute kidney injury in hospitalized diabetic ketoacidosis patients. BMC Nephrol.

[4] Munro JF, Campbell IW, McCuish AC, Duncan LJ (1973). Euglycaemic diabetic ketoacidosis. Br Med J.

[5] Plewa MC, Bryant M, King-Thiele R (2024). Euglycemic Diabetic Ketoacidosis. https://pubmed.ncbi.nlm.nih.gov/32119457/.

[6] Huang SK, Huang CY, Lin CH (2020). Acute kidney injury is a common complication in children and adolescents hospitalized for diabetic ketoacidosis. PLoS One.

[7] Nasa P, Chaudhary S, Shrivastava PK, Singh A (2021). Euglycemic diabetic ketoacidosis: a missed diagnosis. World J Diabetes.

